# The Early Diagnosis of Consumption

**Published:** 1904-02-20

**Authors:** 


### THE EARLY DIAGNOSIS OF CONSUMPTION.

In the light of accumulated experience in the modern treatment of pulmonary tuberculosis it has become a matter of supreme importance to recognise the condition at the earliest possible moment. The success or failure of treatment will depend largely upon this point—whether the patient be treated in a sanatorium or not. As Dr. Cattle[1] remarks, sanatorium treatment is no panacea; even incipient and seemingly favourable cases sometimes become worse in spite of every effort. Yet, speaking generally, the earliest cases are those which do the best. Dr. Cattle deprecates the modern tendency towards leaving the diagnosis to bacteriologists. He points out that although the presence of tubercle bacilli in the sputum is a certain proof of the presence of tuberculosis in the lungs or some part of the respiratory passages, yet in early cases, where evidence is most wanted, bacilli often cannot be discovered, and their absence from the sputum cannot be held to negative a diagnosis of pulmonary tuberculosis. It is well, therefore, not to waste valuable time in repeated attempts to obtain a positive bacteriological result, in the presence of appreciable physical signs of the disease.

In considering the physical signs it must be borne in mind that slight differences between the apices of the two lungs are frequently to be met with even in quite healthy individuals. At the normal right apex there may be slight dulness on percussion, more audible breath-sounds, and more obvious vocal fremitus and vocal resonance. These differences are due to the fact that the right bronchus is wider, shorter, and more horizontal than the left, while it enters the lung at a higher level and gives off the bronchus to the upper lobe nearer the trachea than that for the left upper lobe is given off. For these reasons the glottic sounds are better conducted to the right apex. These peculiarities may cause error in two directions; either slightly more audible, but normal, sounds at the right apex may be regarded as pathological, or commencing signs of consolidation may be regarded as being within the physiological limit. As the right apex is more frequently affected with tuberculosis than the left, the difficulty in discriminating between health and disease is enhanced. If, on the other hand, there is slightl dulness at the left apex, if the breath-sounds, voca resonance and fremitus equal or exceed in intensity those on the right, there is almost certainly disease. Error is liable in left-handed patients, for in them the left apex is commonly less resonant than the right.

In addition to examining the sputum for bacilli and carefully investigating the physical signs another means of diagnosis which is occasionally of use is the injection of Koch's tuberculin. The patient's history and present general state of health must also be duly considered, and it must be remembered that true hæmoptysis in the great majority of instances, even in the absence of physical signs, signifies tuberculosis, though it is necessary to exclude the possibility of the blood coming from a lesion of the nose, mouth, pharynx, larynx, or stomach.

[1] Practitioner, February 1904.

